# Development and Validation of the CARRA-VID Prognostic Score: C Reactive Protein to Albumin Ratio, Red Blood Cell Distribution Width and Age-Based Score for Prognostication of Hospitalized COVID-19 Patients

**DOI:** 10.3390/v17050629

**Published:** 2025-04-27

**Authors:** Marko Lucijanic, Nevenka Piskac Zivkovic, Nikolina Busic, Josip Stojic, Armin Atic, Lovorka Derek, Ivan Krecak, Bruno Barsic, Ivica Luksic

**Affiliations:** 1 Division of Hematology, Internal Medicine Department, University Hospital Dubrava, 10000 Zagreb, Croatia; 2 Scientific Research and Translational Medicine Department, University Hospital Dubrava, 10000 Zagreb, Croatia; 3 School of Medicine, University of Zagreb, 10000 Zagreb, Croatia; 4 Division of Pulmonology, Internal Medicine Department, University Hospital Dubrava, 10000 Zagreb, Croatia; 5 Faculty of Pharmacy and Biochemistry, University of Zagreb, 10000 Zagreb, Croatia; 6 Internal Medicine Department, University Hospital Dubrava, 10000 Zagreb, Croatia; 7 Division of Gastroenterology, Hepatology and Clinical Nutrition, Internal Medicine Department, University Hospital Dubrava, 10000 Zagreb, Croatia; 8 Division of Nephrology, Arterial Hypertension, Dialysis and Transplantation, Internal Medicine Department, University Hospital Center Zagreb, 10000 Zagreb, Croatia; 9 Clinical Department for Laboratory Diagnostics, University Hospital Dubrava, 10000 Zagreb, Croatia; 10 School of Medicine, Catholic University of Croatia, 10000 Zagreb, Croatia; 11 Internal Medicine Department, General Hospital of Sibenik-Knin County, 22000 Sibenik, Croatia; 12 Faculty of Medicine, University of Rijeka, 51000 Rijeka, Croatia; 13 University of Applied Sciences, 22000 Sibenik, Croatia; 14 Maxillofacial Surgery Department, University Hospital Dubrava, 10000 Zagreb, Croatia

**Keywords:** thromboinflammation, SARS-CoV-2, cytokine storm, prognostication, mortality, coronavirus disease 2019

## Abstract

Patients hospitalized due to coronavirus disease 2019 (COVID-19) usually present with severe or critical intensity of symptoms, accompanied by a marked systemic inflammatory response. Classical inflammatory biomarkers, C-reactive protein (CRP), albumin, and red blood cell distribution width (RDW) have previously been reported to be prognostic in hospitalized COVID-19 patients. We performed a retrospective analysis of two large cohorts (2305 and 2328 patients, respectively) of consecutive hospitalized COVID-19 patients with mostly severe and critical symptoms admitted to the tertiary referral center to develop and validate a prognostic score for 30-day mortality based on CRP-to-Albumin-Ratio (CAR), RDW, and age (termed CARRA-VID score). We identified 6 prognostic categories: very low, low, intermediate-1, intermediate-2, high, and very high risk, with corresponding 30-day mortality rates of 2.7%, 10.7%, 30.9%, 47.1%, 61.9%, and 89.7%, respectively. Effective risk stratification was validated in an independent cohort of patients and remained independent of the World Health Organization-defined disease severity and other commonly utilized risk scores. Additional analyses evaluated the score across different time periods dominated by distinct viral variants. We also present a simplified 3-tiered version of the score. A Microsoft Excel Workbook containing the score calculator is provided.

## 1. Introduction

Coronavirus disease 2019 (COVID-19), caused by severe acute respiratory syndrome coronavirus 2 (SARS-CoV-2), is a systemic inflammatory disease that affects multiple organ systems [1]. Although the majority of patients experience only mild or moderate symptoms, up to 15–20% of those infected prior to the availability of vaccines developed severe or critical disease [2], often characterized by respiratory deterioration that could progress to multiorgan failure and death. The underlying mechanism has been identified as thromboinflammation [3], a systemic response to SARS-CoV-2 infection that leads to cytokine storm, defined as the overproduction and uncontrolled release of pro-inflammatory cytokines [4]. The primary mediators of the cytokine storm, which contribute to acute respiratory distress syndrome (ARDS) and multiorgan damage, are interleukins (IL) 1, 6, and 17, as well as tumor necrosis factor alpha (TNF alpha) [5]. These mediators drive an acute-phase inflammatory response in the liver, stimulating the production of C-reactive protein (CRP) while shifting protein metabolism towards increased production of other inflammatory mediators and reduced synthesis of albumin [6].

Erythropoiesis is a highly active metabolic process [7] that provides the body with an adequate number of red blood cells, the most abundant subgroup of blood cells, measured on the order of 10^12^, compared to 10^9^ for other subtypes of cells [8]. It is highly sensitive to various nutritional, inflammatory, and metabolic disturbances, which are readily reflected in the quality of erythropoiesis and which can be evaluated through red blood cell parameters [9]. Anisocytosis, i.e., unequal size of erythrocytes, can be assessed through red blood cell distribution width (RDW), a parameter that quantifies the degree of anisocytosis [10].

CRP and albumin are classic biomarkers of inflammation, widely used in various clinical contexts for both diagnostic and prognostic purposes, including septic, malignant, and inflammatory diseases [11,12,13,14,15]. RDW can be used for the same purpose in numerous clinical settings [16] and is significantly affected by chronic inflammatory comorbidities. Higher age and the presence of comorbidities substantially increase the mortality risk among COVID-19 patients [17]. CRP, albumin, and RDW have also been shown to be deranged and predictive of outcomes in severely ill COVID-19 patients [18,19]. Since these biomarkers are readily accessible from routine blood tests at the time of hospital admission, they hold great potential for providing useful clinical information. Thus, we aimed to integrate these parameters, along with patient age, into a comprehensive prognostic score that may assist in early clinical decision-making. We termed this score the CARRA-VID score (CRP to Albumin Ratio, Red blood cell distribution width, and age-based score for prognostication of hospitalized COVID-19 patients).

## 2. Materials and Methods

### 2.1. Patients and Methods

We retrospectively analyzed two cohorts of consecutively hospitalized COVID-19 patients from a tertiary-level institution for COVID-19 patients, University Hospital Dubrava, Zagreb, Croatia, who had available data on CRP, albumin, RDW, age, and clinical outcomes. Patients were treated in the period from March 2020 to March 2021 for the development cohort (Nm: 2305 patients; Nm—number) and from March 2021 to June 2022 for the validation cohort (Nm: 2328 patients). During the period of the pandemic, the hospital was completely repurposed to serve as a regional referral center for the most severe cases of COVID-19 and for SARS-CoV-2-positive patients who presented with other medical/surgical/neurological emergencies. All patients were ≥18 years of age, were of White race, and tested positive on polymerase chain reaction (PCR) or rapid antigen COVID-19 test prior to hospital admission. We evaluated only the first COVID-19-related hospitalizations for each patient.

Demographic, clinical, laboratory, and data on the course of treatment and outcomes were obtained from the written and electronic medical records and are a part of a hospital registry project registered at clinicaltrials.gov (Nm: NCT05151094). The study was approved by the Institutional Review Board (Nm: 2021/2503-04), which waived the need for informed consent due to the retrospective nature of the study.

Severity of COVID-19 was graded according to the World Health Organization (WHO) criteria into mild, moderate, severe, and critical [20]. Intensity of symptoms was graded using the Modified Early Warning Score (MEWS) [21]. Patients were treated according to the contemporary guidelines. Comorbidities were evaluated as separate entities and as a cumulative comorbidity burden quantified using the Charlson comorbidity index [22]. Functional status was graded using the Eastern Cooperative Oncology Group (ECOG) scale [23]. CRP, albumin, and RDW were obtained among other laboratory parameters at the time of hospital admission using the AU5800 analyzer (Beckman Coulter, Tokyo, Japan) for biochemical analysis and the Advia 2120i counter for complete blood count parameters (Siemens Medical Solutions Diagnostics Pte Ltd., Swords, Ireland).

Mortality was assessed from the start of hospital admission until 30 days. CURB-65 (confusion, urea, respiratory rate, blood pressure, and 65 years of age) [24], VACO-index (Veterans Health Administration COVID-19) [25], and 4C mortality score [26] were used as standard prognostic scores.

### 2.2. Statistical Methods

Normality of distribution of numerical variables was assessed using the Kolmogorov–Smirnov test. Due to non-normal distribution in most cases, numerical variables were summarized as median and interquartile range (IQR) and were compared between groups using the Kruskal–Wallis ANOVA test/the Mann–Whitney U test. Categorical variables were summarized as ratios and percentages and were compared between groups using the chi-squared test. The Jonckheere—Terpstra test for trend for continuous variables and the chi-squared test for trend for categorical variables were used to evaluate trends of increase or decrease in investigated parameters over categories of CARRA-VID score. Survival analysis was based on the method by Kaplan and Meier [27]. The Cox regression analysis was used to assess the independent contribution of different variables and to obtain hazard ratios with respective 95% confidence intervals (CI) for investigated parameters. Harrell’s C index, obtained from the respective Cox regression models, was used as a measure of the prognostic accuracy of the investigated models, describing concordance between predicted and observed outcomes (probability of correctly predicted outcomes). *p* values < 0.05 were considered statistically significant. All analyses were carried out using the MedCalc statistical software version 23.2.1 (MedCalc Software Ltd., Ostend, Belgium). The custom-made Microsoft Excel (Microsoft, Redmond, Washington, DC, USA) workbook-based calculator was created for novel score calculation and is provided as a Supplementary File (Supplementary File S1).

### 2.3. The Process of Score Development

We split the hospital registry dataset with available data on parameters of interest into two parts, termed development and validation cohorts, respectively. Tables and Figures used through the development process are provided in Supplementary File S2. Using the development cohort of patients, we verified mutually independent prognostic properties of higher CRP, lower albumin, higher RDW, and older age for worse survival in a multivariable Cox regression model (Supplementary Table S1). We further stratified patients on previously defined and published cut-off points for CRP to albumin ratio (CAR) [18], RDW [19], and age [17], developed in the same patient cohort, with care that rounded cut-off points are used and certain risk categories defined by these cut-off points are adequately represented by a sufficient number of patients. Each category was given a certain number of points corresponding to the observed hazard ratios (HR) for 30-day mortality obtained from the Cox regression analysis (Supplementary Table S2); mortality rates associated with each category of CAR, RDW, and age are shown in Supplementary Figures S1–S3.

The following categories were established for CAR (when CRP is measured in mg/L and albumin in g/L): <1 (assigned 0 points), ≥1 to <3 (assigned 2 points), ≥3 to <6 (assigned 3 points), and ≥6 (assigned 5 points). The following categories were established for RDW (measured as RDW-CV in percentage points): <13% (assigned 0 points), ≥13% to <14% (assigned 1 point), ≥14% to <15% (assigned 2 points), ≥15% to <16% (assigned 3 points), and ≥16% (assigned 4 points). The following categories were established for age (in years): <60 years (assigned 0 points), ≥60 to <70 years (assigned 1 point), ≥70 to <85 years (assigned 3 points), and ≥85 years (assigned 5 points). Points were summarized for each patient, who could achieve from 0 to 14 points in total. Distribution of 30-day mortality rates according to the cumulative score obtained according to the above-described procedure is shown in Supplementary Figure S4.

We further combined cumulative points into groups based on similar mortality rates observed, yielding a total of six risk categories of the novel CARRA-VID score: very low risk (0–2 cumulative points), low risk (3–4 cumulative points), intermediate-1 risk (5–7 points), intermediate-2 (8 points), high risk (9–12 points), and very high risk (13–14 points). Due to low proportions of patients in very low, intermediate-2, and very high-risk categories, scores could be further simplified into three tiers by combining low, intermediate, and high-risk categories together, creating a simplified CARRA-VID (sCARRA-VID) score.

The findings were replicated in the validation cohort of patients, confirming the robustness of the novel score.

## 3. Results

### 3.1. Overview of the Development and Validation Cohorts of Patients

A total of 2305 patients in the development and 2328 patients in the validation cohort were analyzed. The characteristics of patients in both cohorts are summarized in Table 1. The development cohort consisted almost exclusively of patients infected with the Alpha SARS-CoV-2 strain, with a median age of 73 years and proportions of severe and critical symptoms on presentation of 69.8% and 16%, respectively. The validation cohort consisted of patients infected with Beta/Gamma, followed by Delta and Omicron strains of SARS-CoV-2, with a median age of 70 years and proportions of severe and critical symptoms on presentation of 70.1% and 19.3%, respectively. Patients in the validation cohort were significantly younger, had lower comorbidity burden, and were less likely to have additional co-infection, but had more severe disease presentation on average (*p* < 0.05 for all analyses). Although CRP (median 91.1 mg/L vs. 93.5 mg/L) and albumin levels (median 32 g/L vs. 31.5 g/L) were similar between cohorts, patients in the validation cohort presented with lower RDW values (median 14.1% vs. 13.9%, *p* < 0.001).

### 3.2. Overview of the CARRA-VID Score in the Development Cohort

In the development cohort of patients, the median number of cumulative CARRA-VID points was 7, IQR (5–9). Median 2, 2, and 3 points were assigned for CAR, RDW, and age, respectively. A total of 150 (6.5%) presented with very low, 384 (16.7%) with low, 901 (39.1%) with intermediate-1, 225 (9.8%) with intermediate-2, 606 with high, and 39 (1.7%) with very high risk categories. Due to low proportions of patients in very low, intermediate-2, and very high risk categories, patients were further combined into more robust categories of sCARRA-VID score, where a total of 534 (23.2%) patients presented with low risk, 1126 (49.9%) with intermediate risk, and 645 (28%) with high-risk disease.

Stratification of patients’ characteristics by the levels of sCARRA-VID score is presented in Table 2. Patients belonging to the higher risk categories were more likely to be of older age, female sex (although male sex was more prevalent in all three risk categories), to have more rapid deterioration from the development of first symptoms, to have worse functional status and higher intensity of COVID-19-related symptoms on admission, to more often have pneumonia and require oxygen therapy, to more often have additional co-infections on admission, to have higher comorbidity burden, and to have worse thromboinflammatory laboratory profiles (*p* < 0.05 for all analyses).

### 3.3. Prognostic Properties of the CARRA-VID Score in the Development and Validation Cohorts

**Figure 1:**
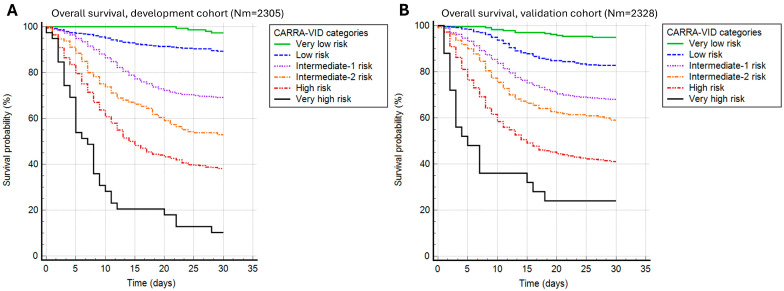
A 30-day post-hospital admission survival stratified by the CARRA-VID prognostic score categories in (**A**) development and (**B**) validation cohorts of patients.

The CARRA-VID score was able to significantly discriminate 30-day survival in both development and validation cohorts of patients (*p* < 0.001 for both contexts), as shown in Figure 1. Harrell’s C index was 0.712 for the development and 0.722 for the validation cohort of patients. The score was able to accurately discriminate 6 prognostically distinct categories of patients experiencing different clinical courses during the 30-day post-hospital admission period.

Observed 30-day mortality rates in the development cohort were 2.7% for the very low, 10.7% for the low, 30.9% for the intermediate-1, 47.1% for the intermediate-2, 61.9% for the high, and 89.7% for the very high-risk category. Very similar prognostic discrimination was present in the validation cohort, with 30-day mortality rates of 5.1%, 17.2%, 31.9%, 40.9%, 58.9%, and 76% for very low, low, intermediate-1, intermediate-2, high, and very high-risk categories.

**Figure 2:**
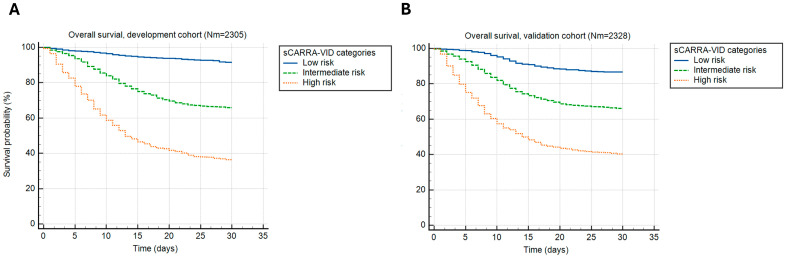
A 30-day post-hospital admission survival stratified by the simplified CARRA-VID (sCARRA-VID) prognostic score categories in (**A**) development and (**B**) validation cohorts of patients.

Similar could be shown for sCARRA-VID categories that provided 3-tiered prognostic stratification, with prognostic accuracy (Harrell’s C index) of 0.696 and 0.712 for the development and validation cohorts, respectively, as shown in Figure 2.

Observed 30-day mortality rates in the development cohort were 8.4% for the low, 34.1% for the intermediate, and 63.6% for the high-risk category. The 30-day mortality rates in the validation cohort were 13.3%, 33.8%, and 59.7% in the low, intermediate, and high-risk categories, respectively.

### 3.4. Comparison of the CARRA-VID Score to Other Established Prognostic Systems

We further analyzed a series of multivariable Cox regression analysis models comparing the CARRA-VID score to other established scores used for prognostication of COVID-19 patients: CURB-65, 4C mortality score, and VACO index, as well as the WHO-defined COVID-19 severity stratification system. CARRA-VID retained independent prognostic properties regardless of all of the investigated risk stratification systems. Regression models are presented in Supplementary File S2, Supplementary Table S3.

Compared to other prognostic models, CARRA-VID and sCARRA-VID seem to possess a similar degree of prognostic accuracy (Harrell’s C index of 0.708, 95% CI (0.692–0.724) for CARRA-VID and 0.692, 95% CI (0.676–0.708) for sCARRA-VID) as CURB-65 (0.714, 95% CI (0.698–0.730)) and 4C mortality score (0.709, 95% CI (0.694–0.724)), and higher accuracy of estimation than VACO-index (0.654, 95% CI (0.637–0.671)) and the WHO-defined COVID-19 severity stratification system (0.64, 95% CI (0.626–0.655)).

### 3.5. Usefulness of the CARRA-VID Score over Different Time Periods Dominated by Distinct Viral Strains

We further analyzed how the CARRA-VID score performed among different subgroups of patients defined by periods of the pandemic dominated by distinct viral strains.

**Figure 3:**
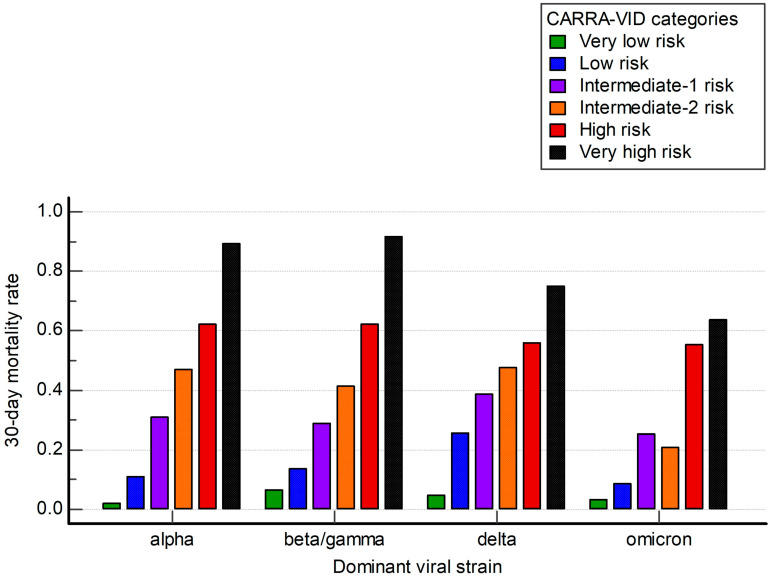
A 30-day post-hospital admission mortality stratified by the CARRA-VID prognostic score categories over periods dominated by specific SARS-CoV-2 viral strains.

The CARRA-VID prognostic score retained prognostic accuracy over investigated periods, as shown in Figure 3. Observed Harrell’s C index values were 0.707, 0.718, 0.659, and 0.717 for periods dominated by alpha, beta/gamma, delta, and omicron viral strains, respectively.

## 4. Discussion

As we show in the current study, combining information on CRP, albumin, RDW, and age in hospitalized COVID-19 patients results in a comprehensive, clinically relevant prognostic score that incorporates different aspects of thromboinflammation and chronic comorbidities. We have developed and validated a novel CARRA-VID prognostic score in two large cohorts of consecutive hospitalized COVID-19 patients (development and validation cohorts), demonstrating its potent prognostic properties.

Acutely ill COVID-19 patients are often unable to provide accurate medical history, and clinical and laboratory parameters often guide medical decision-making. Parameters derived from a laboratory workup enable unique insight into the biology and pre-defined risks of SARS-CoV-2 infection. The major strength of the CARRA-VID score is its sole reliance on objective laboratory measurements of CRP, albumin, and RDW, obtained at the time of patient presentation. This novel score may have wide applicability, since its components are easily obtainable at the time of hospital admission in the majority of acutely ill COVID-19 patients. Incorporated elements are established parameters of inflammation that may be caused by the host response to SARS-CoV-2 infection but may also be additionally supported by the presence of chronic metabolic and other comorbidities [18]. CRP, albumin, and RDW seem to depict different aspects of thromboinflammation and therefore seem to provide mutually independent prognostic information. RDW, among investigated parameters, seems to be substantially affected by specific comorbidities predating SARS-CoV-2 infection and seems to be similarly prognostic of worse outcome regardless of the presence of anemia [28]. All of the investigated parameters have been previously shown to be prognostic in the contexts of diseases with strong inflammatory backgrounds prior to the occurrence of the COVID-19 pandemic [13,14,29] and were shown to have the potential to further improve commonly used COVID-19 risk scores [18,30].

One of the major strengths of the novel CARRA-VID score is its ability to provide six different risk categories for 30-day mortality, enabling precise stratification of patients at the time of hospital admission. All of the six risk categories follow a distinct clinical course, which enables a higher level of prognostic information than provided by other scores and the WHO-defined disease severity stratification. When compared to other prognostic systems commonly used in COVID-19 patients, CARRA-VID retains independent statistical significance, suggesting its potent and clinically useful potential on top of other contemporary risk stratification systems.

Findings of potent risk stratification using the CARRA-VID prognostic score seem to be stable during different time periods dominated by different viral strains. However, it can be seen that during the time period dominated by the Omicron SARS-CoV-2 viral strain, there seems to be a loss of prognostic discrimination between subgroups of lower, intermediate, and higher risk, respectively, prompting the use of simplified scores.

Limitations of our work are single-center experience, retrospective study design, and inability to control for a number of potentially unmeasured confounders that may affect interpretation of final results. No causality of provided findings can be inferred due to the retrospective nature of the study. Nevertheless, the CARRA-VID score represents a unique way of quantifying CRP, albumin, and RDW derangements into the prognostic score.

## 5. Conclusions

The CARRA-VID prognostic score, developed and validated in two large consecutive cohorts of hospitalized COVID-19 patients, respectively, provides an easy and accurate method for 30-day survival prognostication. The risk stratification obtained using the CARRA-VID prognostic score appears to be independent of other contemporary prognostic scores. Additionally, we have developed an MS Excel Workbook that enables the calculation of the CARRA-VID score on an individual patient basis. We hope that a simple and easily obtainable score like CARRA-VID will aid in clinical decision-making and facilitate the early recognition and treatment of the most vulnerable COVID-19 patients.

## Figures and Tables

**Table 1:** Patients’ characteristics in the development and validation cohorts of patients.

	Development Cohort (N = 2305)	Validation Cohort (N = 2328)	*p* Value
**Age** (years)	73 (64–81)	70 (60–79)	<0.001 *
**Male sex**	1323 (57.4%)	1287 (55.3%)	0.147
**ECOG status on admission**	3 (1–4)	3 (1–4)	0.872
**MEWS score**	2 (1–4)	3 (1–4)	<0.001 *
**COVID-19 severity**			
Mild	217 (9.4%)	121 (6.8%)	<0.001 *
Moderate	110 (4.8%)	68 (3.8%)	
Severe	1609 (69.8%)	1252 (70.1%)	
Critical	369 (16%)	345 (19.3%)	
**Co-infection on admission**	335 (14.5%)	187 (10.1%)	<0.001 *
**Charlson comorbidity index**	4 (3–6)	4 (2–5)	<0.001 *
**CRP** (mg/L)	91.1 (42.9–153.8)	93.5 (43.3–158.9)	0.334
**Albumin** (g/L)	32 (28–35)	31.5 (29–34)	0.5271
**RDW** (%)	14.1 (13.4–15.3)	13.9 (13.3–15)	<0.001 *
**Dominant viral strain**			
Alpha	2259 (98%)	-	<0.001 *
Beta/Gamma	46 (2%)	1155 (49.6%)	
Delta	-	816 (35.1%)	
Omicron	-	357 (15.3%)	

* Statistically significant at level *p* < 0.05/Data are presented as median and interquartile range for numerical variables and as frequency and percentage for categorical variables./The Mann–Whitney U test for numerical and the chi-squared test for categorical variables were used./Abbreviations: ECOG—Eastern Cooperative Oncology Group; MEWS—Modified Early Warning Score; COVID-19—Coronavirus disease 2019; CRP—C-reactive protein; RDW—red blood cell distribution width.

**Table 2:** Patients’ characteristics stratified according to the simplified CARRA-VID score categories.

Development Cohort	sCARRA-VID Low Risk	sCARRA-VID Intermediate Risk	sCARRA-VID High Risk	*p* Value
**Number of patients**	534 (23.2%)	1126 (48.9%)	645 (28%)	-
**Age** (years)	59 (51–67)	73 (65–80)	82 (75–87)	<0.001 *
**Male sex**	339 (63.5%)	641 (56.9%)	343 (53.2%)	<0.001 *
**Day of disease on admission**	6 (1–9)	5 (1–9)	4 (1–8)	0.001 *
**ECOG status on admission**	1 (1–3)	2 (1–3)	3 (2–4)	<0.001 *
**Pneumonia**	429 (80.3%)	1024 (90.9%)	619 (96%)	<0.001 *
**Oxygen therapy**	382 (71.5%)	957 (85%)	599 (92.9%)	<0.001 *
**MEWS score**	2 (1–3)	2 (1–4)	3 (1–4)	<0.001 *
**COVID-19 severity**				
Mild	97 (18.2%)	95 (8.4%)	25 (3.9%)	<0.001 *
Moderate	38 (7.1%)	55 (4.9%)	17 (2.6%)	
Severe	351 (65.7%)	803 (71.3%)	455 (70.5%)	
Critical	48 (9%)	173 (15.4%)	148 (22.9%)	
**Co-infection on admission**	35 (6.6%)	160 (14.2%)	140 (21.7%)	<0.001 *
**Charlson comorbidity index**	2 (1–4)	4 (3–6)	6 (4–7)	<0.001 *
**Arterial hypertension**	298 (55.8%)	831 (73.8%)	491 (76.1%)	<0.001 *
**Diabetes mellitus**	134 (25.1%)	383 (34%)	214 (33.2%)	0.005 *
**Hyperlipoproteinemia**	117 (21.9%)	285 (25.3%)	152 (23.6%)	0.569
**Obesity**	170 (31.8%)	338 (30%)	143 (22.2%)	<0.001 *
**Cong. heart failure**	42 (7.9%)	177 (15.7%)	158 (24.5%)	<0.001 *
**Atrial fibrillation**	30 (5.6%)	205 (18.2%)	179 (27.8%)	<0.001 *
**Coronary artery disease**	76 (14.2%)	183 (16.3%)	107 (16.6%)	0.285
**Peripheral artery disease**	23 (4.3%)	88 (7.8%)	62 (9.6%)	<0.001 *
**Previous CVI**	31 (5.8%)	125 (11.1%)	99 (15.3%)	<0.001 *
**Previous myocardial inf.**	43 (8.1%)	109 (9.7%)	64 (9.9%)	0.288
**Chr. kidney disease**	38 (7.1%)	153 (13.6%)	104 (16.1%)	<0.001 *
**COPD**	33 (6.2%)	88 (7.8%)	54 (8.4%)	0.165
**Chronic liver disease**	21 (3.9%)	44 (3.9%)	18 (2.8%)	0.273
**Liver cirrhosis**	9 (1.7%)	19 (1.7%)	10 (1.6%)	0.849
**Active malignancy**	38 (7.1%)	127 (11.3%)	108 (16.7%)	<0.001 *
**Metastatic malignancy**	21 (3.9%)	85 (7.5%)	6 (10.2%)	<0.001 *
**Dementia**	31 (5.8%)	180 (16%)	217 (33.6%)	<0.001 *
**Alcohol use**	33 (6.2%)	83 (7.4%)	26 (4%)	0.093
**Smoking**	46 (8.6%)	76 (6.7%)	19 (2.9%)	<0.001 *
**IL-6** (pg/mL)	22.9 (8.7–65.5)	50.9 (22.1–118.4)	105.5 (47.9–271.5)	<0.001 *
**Procalcitonin** (ng/mL)	0.09 (0.05–0.21)	0.21 (0.09–0.63)	0.59 (0.22–2.69)	<0.001 *
**WBC** (×10^9^/L)	7.45 (5.3–10.2)	7.8 (5.6–11)	9.3 (6.7–13.2)	<0.001 *
**Abs. lymphocytes** (×10^9^/L)	1.0 (0.7–1.5)	0.8 (0.53–1.2)	0.7 (0.5–1.0)	<0.001 *
**Hemoglobin** (g/L)	135 (122–144)	127 (113–140)	119 (104–133)	<0.001 *
**Platelets** (×10^9^/L)	222.5 (173–305)	218 (159–297)	219 (156–298)	0.270
**CRP** (mg/L)	34.1 (14–82.8)	88.4 (46.5–138.6)	154.4 (93.9–215.1)	<0.001 *
**Ferritin** (µg/L)	634 (309–1182)	725 (416–1291)	741 (411–1351)	0.001 *
**D-dimers** (mg/L FEU)	0.82 (0.51–1.88)	1.37 (0.77–3.21)	2.21 (1.15–4.25)	<0.001 *
**CKD-EPI eGFR** (ml/min/1.73 m^2^)	92.2 (73.9–102.8)	71.9 (48.2–90)	54.2 (32.6–77.4)	<0.001 *
**LDH** (U/L)	288 (213–388.8)	355 (258–482)	374.5 (274–514)	<0.001 *
**AST** (U/L)	36 (25–56)	41.5 (29–64)	45 (30–71)	<0.001 *
**ALT** (U/L)	33 (21–56)	31 (19–52)	28 (17–46)	<0.001 *
**GGT** (U/L)	45 (24–89.7)	43 (26–87)	40.5 (22–80)	0.183
**ALP** (U/L)	67 (53–86)	70 (55–96)	80 (59–110)	<0.001 *
**Total bilirubin** (µmol/L)	10.5 (8.2–14.2)	11.2 (8.6–15.6)	12 (8.7–17.7)	<0.001 *
**Albumin** (g/L)	35 (32–38)	32 (29–34)	29 (26–32)	<0.001 *
**PT** (%)	104 (95–111)	101 (90–109)	94 (84–104)	<0.001 *

* statistically significant at level *p* < 0.05/Data are presented as median and interquartile range for numerical variables and as frequency and percentage for categorical variables./The Jonckheere—Terpstra test for trend for numerical and the chi-squared test for trend for categorical variables were used to evaluate trends of increase or decrease in investigated parameters./Abbreviations: ECOG—Eastern Cooperative Oncology Group; MEWS—modified early warning score; Chr.—chronic; Cong.—congestive; CVI—cerebrovascular insult; COPD—chronic obstructive lung disease; WBC—white blood cells; Abs.—absolute; CRP—C-reactive protein; CKD-EPI—Chronic Kidney Disease Epidemiology Collaboration; eGFR—estimated glomerular filtration rate; LDH—lactate dehydrogenase; AST—aspartate aminotransferase; ALT—alanine aminotransferase; GGT—gamma-glutamyl transferase; ALP—alkaline phosphatase; PT—prothrombin time.

## Data Availability

The data presented in this study are available on request from the corresponding author (the data are not publicly available due to privacy or ethical restrictions, i.e., individual patients can be identified from the dataset due to specific disease, timing, and location context).

## Supplementary Materials

The following supporting information can be downloaded at: https://www.mdpi.com/article/10.3390/v17050629/s1, Supplementary File S1, MS Excel Workbook based calculator for CARRA-VID score; Supplementary File S2, an overview of the score development and evaluation process. Table S1: The Cox regression model for 30-days survival mutually comparing CRP, albumin, RDW and age, tested in the development cohort. Table S2: Hazard ratios and assigned points for C-reactive protein to Albumin Ratio (CAR), RDW and age categories, tested in the development cohort. Figure S1: The 30-day mortality rates for different C reactive protein to Albumin Ratio (CAR) categories, tested in the development cohort. The orange color represents the proportion of deceased patients, while the blue color represents the proportion of surviving patients. Figure S2: The 30-day mortality rates for different red blood cell distribution width (RDW) categories, tested in the development cohort. The orange color represents the proportion of deceased patients, while the blue color represents the proportion of surviving patients. Figure S3: The 30-day mortality rates for different age categories, tested in the development cohort. The orange color represents the proportion of deceased patients, while the blue color represents the proportion of surviving patients. Figure S4: The 30-day mortality rates corresponding to each point of the cumulative CARRA-VID score, tested in the development cohort. The orange color represents the proportion of deceased patients, while the blue color represents the proportion of surviving patients. Figure S5: Histograms of distribution of (A) C reactive protein (CRP), (B) albumin, (C) CRP to albumin ratio (CAR), (D) red blood cell distribution width (RDW) and (E) age. Table S3: Overview of the Cox regression analysis models comparing the CARRA-VID to other established prognostic scores. Table S4: Overview of the distribution of investigated variables used for the creation of the CARRA-VID score in the development cohort.

## References

[1] Ciotti M., Angeletti S., Minieri M., Giovannetti M., Benvenuto D., Pascarella S., Sagnelli C., Bianchi M., Bernardini S., Ciccozzi M. (2019). COVID-19 Outbreak: An Overview. Chemotherapy.

[2] Wu Z., McGoogan J.M. (2020). Characteristics of and Important Lessons From the Coronavirus Disease 2019 (COVID-19) Outbreak in China: Summary of a Report of 72,314 Cases From the Chinese Center for Disease Control and Prevention. Jama.

[3] Obeagu E.I., Obeagu G.U. (2024). Thromboinflammation in COVID-19: Unraveling the interplay of coagulation and inflammation. Medicine.

[4] Montazersaheb S., Hosseiniyan Khatibi S.M., Hejazi M.S., Tarhriz V., Farjami A., Ghasemian Sorbeni F., Farahzadi R., Ghasemnejad T. (2022). COVID-19 infection: An overview on cytokine storm and related interventions. Virol. J..

[5] Park M.D. (2020). Macrophages: A Trojan horse in COVID-19?. Nat. Rev. Immunol..

[6] Robinson M.W., Harmon C., O’Farrelly C. (2016). Liver immunology and its role in inflammation and homeostasis. Cell. Mol. Immunol..

[7] Cha H.J. (2024). Erythropoiesis: Insights from a genomic perspective. Exp. Mol. Med..

[8] Lucijanic M., Krecak I. (2023). The Complete Blood Count: Increasing Its Precision and Impact. Ann. Intern. Med..

[9] Yang C., Suda T. (2025). Microenvironmental dynamics in steady-state and stress erythropoiesis. Blood Sci..

[10] Patel K.V., Semba R.D., Ferrucci L., Newman A.B., Fried L.P., Wallace R.B., Bandinelli S., Phillips C.S., Yu B., Connelly S. (2010). Red cell distribution width and mortality in older adults: A meta-analysis. J. Gerontol. A Biol. Sci. Med. Sci..

[11] Lucijanic M., Cicic D., Stoos-Veic T., Pejsa V., Rahelic D., Lucijanic T., Vasilj T., Ivic M., Sedinic M., Kusec R. (2018). Combining information on C reactive protein and serum albumin into the Glasgow Prognostic Score strongly discriminates survival of myelofibrosis patients. Blood Cells Mol. Dis..

[12] Karampela I., Chrysanthopoulou E., Simitsis P., Skyllas G., Christodoulatos G.S., Antonakos G., Kandri E., Armaganidis A., Dalamaga M. (2020). C-reactive protein/albumin ratio as a prognostic biomarker in critically ill septic patients: A prospective study. Eur. Respir. J..

[13] Haider Kazmi S.J., Zafar M.T., Zia B.F., Khalid S.R., Kumar V., Tabassum S., Ali A., Aziz N., Khan N.A., Kumari K. (2022). Role of serum C-reactive protein (CRP)/Albumin ratio in predicting the severity of acute pancreatitis: A retrospective cohort. Ann. Med. Surg..

[14] Sunar İ., Ataman Ş. (2020). Serum C-Reactive Protein/Albumin Ratio in Rheumatoid Arthritis and its Relationship With Disease Activity, Physical Function, and Quality of Life. Arch. Rheumatol..

[15] Wu J., Tan W., Chen L., Huang Z., Mai S. (2018). Clinicopathologic and prognostic significance of C-reactive protein/albumin ratio in patients with solid tumors: An updated systemic review and meta-analysis. Oncotarget.

[16] Lippi G., Plebani M. (2014). Red blood cell distribution width (RDW) and human pathology. One size fits all. Clin. Chem. Lab. Med..

[17] Piskač Živković N., Lucijanić M., Bušić N., Jurin I., Atić A., Andrilović A., Penović T., Domić I., Gnjidić J., Demaria M. (2022). The associations of age, sex, and comorbidities with survival of hospitalized patients with coronavirus disease 2019: Data from 4014 patients from a tertiary-center registry. Croat. Med. J..

[18] Lucijanić M., Stojić J., Atić A., Čikara T., Osmani B., Barišić-Jaman M., Andrilović A., Bistrović P., Zrilić Vrkljan A., Lagančić M. (2022). Clinical and prognostic significance of C-reactive protein to albumin ratio in hospitalized coronavirus disease 2019 (COVID-19) patients: Data on 2309 patients from a tertiary center and validation in an independent cohort. Wien. Klin. Wochenschr..

[19] Lucijanić M., Jordan A., Jurin I., Piskač Živković N., Sorić E., Hadžibegović I., Atić A., Stojić J., Rudan D., Jakšić O. (2022). Red cell distribution width is a potent prognostic parameter for in-hospital and post-discharge mortality in hospitalized coronavirus disease 2019 patients: A registry-based cohort study on 3941 patients. Croat. Med. J..

[20] World Health Organization (2020). Clinical Management of COVID-19: Interim Guidance, 27 May 2020.

[21] Subbe C.P., Kruger M., Rutherford P., Gemmel L. (2001). Validation of a modified Early Warning Score in medical admissions. Qjm.

[22] Charlson M.E., Pompei P., Ales K.L., MacKenzie C.R. (1987). A new method of classifying prognostic comorbidity in longitudinal studies: Development and validation. J. Chronic Dis..

[23] Oken M.M., Creech R.H., Tormey D.C., Horton J., Davis T.E., McFadden E.T., Carbone P.P. (1982). Toxicity and response criteria of the Eastern Cooperative Oncology Group. Am. J. Clin. Oncol..

[24] Lim W.S., van der Eerden M.M., Laing R., Boersma W.G., Karalus N., Town G.I., Lewis S.A., Macfarlane J.T. (2003). Defining community acquired pneumonia severity on presentation to hospital: An international derivation and validation study. Thorax.

[25] King J.T., Yoon J.S., Rentsch C.T., Tate J.P., Park L.S., Kidwai-Khan F., Skanderson M., Hauser R.G., Jacobson D.A., Erdos J. (2020). Development and validation of a 30-day mortality index based on pre-existing medical administrative data from 13,323 COVID-19 patients: The Veterans Health Administration COVID-19 (VACO) Index. PLoS ONE.

[26] Knight S.R., Ho A., Pius R., Buchan I., Carson G., Drake T.M., Dunning J., Fairfield C.J., Gamble C., Green C.A. (2020). Risk stratification of patients admitted to hospital with covid-19 using the ISARIC WHO Clinical Characterisation Protocol: Development and validation of the 4C Mortality Score. Bmj.

[27] Lucijanic M., Skelin M., Lucijanic T. (2017). Survival analysis, more than meets the eye. Biochem. Med. (Zagreb.).

[28] Lucijanic M., Soric E., Sedinic Lacko M., Sabljic A., Krecak I., Bistrovic P., Jordan A., Manola S., Jaksic O., Lucijanic T. (2022). Gradual increase in red cell distribution width is similarly prognostic for in-hospital mortality in both anemic and non-anemic COVID-19 patients. J. Med. Virol..

[29] Al-Rawi Z.S., Gorial F.I., Al-Bayati A.A. (2018). Red Cell Distribution Width in Rheumatoid arthritis. Mediterr. J. Rheumatol..

[30] Jordan A., Trkulja V., Jurin I., Marević S., Đerek L., Lukšić I., Manola Š., Lucijanić M. (2024). Accounting for Red Cell Distribution Width Improves Risk Stratification by Commonly Used Mortality/Deterioration Risk Scores in Adult Patients Hospitalized Due to COVID-19. Life.

