# Supplementary material for: Development and Validation of the CARRA-VID Prognostic Score: C Reactive Protein to Albumin Ratio, Red Blood Cell Distribution Width and Age-Based Score for Prognostication of Hospitalized COVID-19 Patients
Source: Viruses. 2025 Apr 27;17(5):629. doi: 10.3390/v17050629 (PMC12115730; doi:10.3390/v17050629)

Manuscript title: Development and validation of the CARRA-VID prognostic score: C reactive protein to Albumin Ratio, Red blood cell distribution width and Age based score for prognostication of hospitalized COVID-19 patients

Supplementary Appendix B: Graphs and Tables used for development and evaluation of the CARRA-VID score

Supplementary Table S1: The Cox regression model for 30-days survival mutually comparing CRP, albumin, RDW and age, tested in the development cohort.

	**Hazard ratio with 95% Confidence interval**	**P value**
**CRP** (mg/L)	HR 1.004<br>95% CI (1.003-1.004)	<0.001 *
**Albumin** (g/L)	HR 0.93<br>95% CI (0.92-0.95)	<0.001 *
**RDW** (%)	HR 1.09<br>95% CI (1.07-1.13)	<0.001 *
**Age** (years)	HR 1.04<br>95% CI (1.03-1.05)	<0.001 *

*Statistically significant at level P<0.05 / Abbreviations: CRP – C reactive protein, HR – hazard ratio, CI – confidence interval, RDW – red blood cell distribution width.

Supplementary Table S2: Hazard ratios and assigned points for C-reactive protein to Albumin Ratio (CAR), RDW and age categories, tested in the development cohort.

**Variable**	**Cut-off**	**Proportion of pts**	**Observed HR**	**Assigned points**
CAR	<1	20%	Reference category	0
	≥1 to <3	30%	2.48	2
	≥3 to <6	30%	3.43	3
	≥6	20%	5.38	5
RDW	<13%	10%	Reference category	0
	≥13% to <14%	35%	1.62	1
	≥14% to <15%	24%	2.29	2
	≥15% to <16%	15%	2.94	3
	≥16%	15%	3.39	4
Age	<60 years	20%	Reference category	0
	≥60 to <70 years	20%	1.74	1
	≥70 to <85 years	45%	3.51	3
	≥85 years	15%	5.18	5

CAR was calculated as CRP in mg/L divided by Albumin in g/L / Abbreviations: HR – hazard ratio, CAR – C reactive protein to Albumin Ratio, RDW – red blood cell distribution width.

Supplementary Figure S1: The 30-day mortality rates for different C reactive protein to Albumin Ratio (CAR) categories, tested in the development cohort. The orange color represents the proportion of deceased patients, while the blue color represents the proportion of surviving patients.

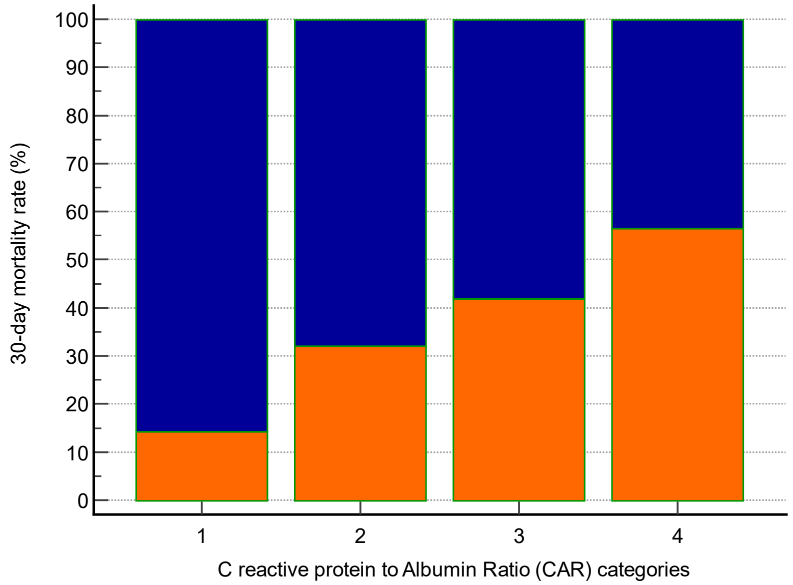

Supplementary Figure S2: The 30-day mortality rates for different red blood cell distribution width (RDW) categories, tested in the development cohort. The orange color represents the proportion of deceased patients, while the blue color represents the proportion of surviving patients.

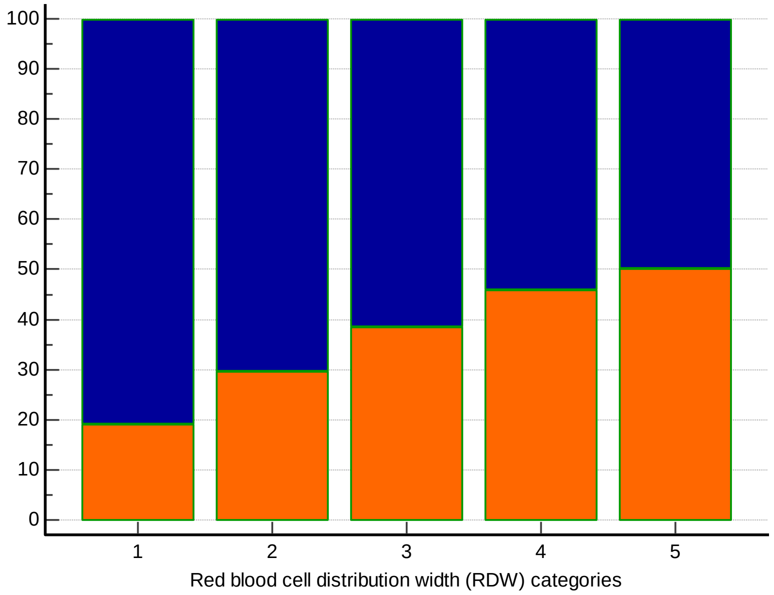

Supplementary Figure S3: The 30-day mortality rates for different age categories, tested in the development cohort. The orange color represents the proportion of deceased patients, while the blue color represents the proportion of surviving patients.

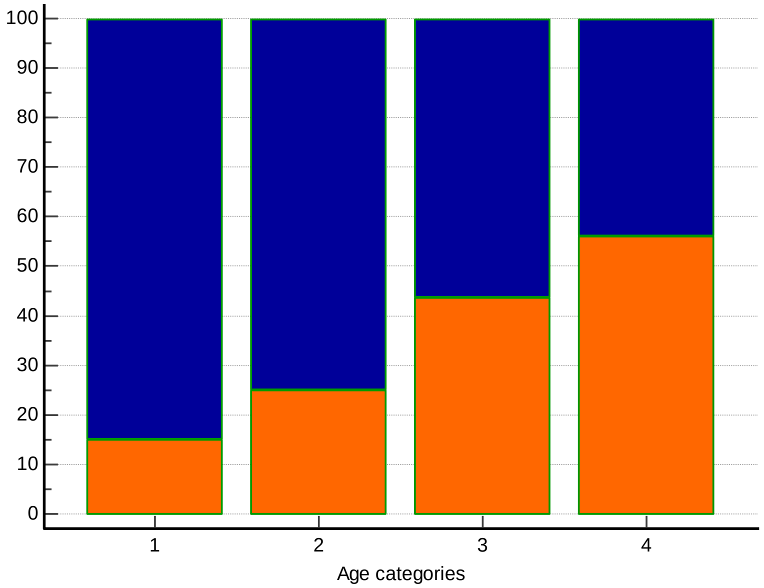

Supplementary Figure S4: The 30-day mortality rates corresponding to each point of the cumulative CARRA-VID score, tested in the development cohort. The orange color represents the proportion of deceased patients, while the blue color represents the proportion of surviving patients.

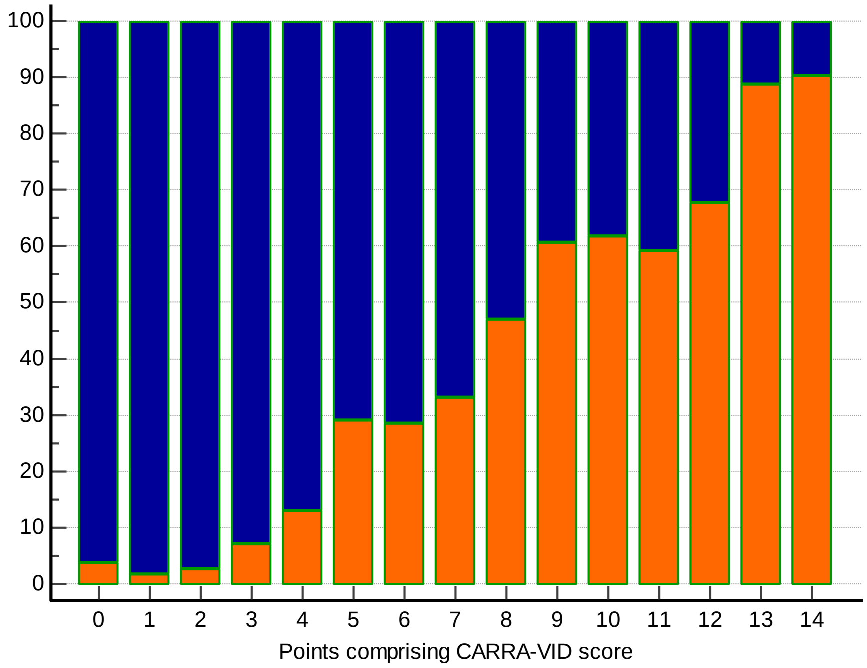

Supplementary Table S3: Overview of the Cox regression analysis models comparing the CARRA-VID to other established prognostic scores.

	**Hazard ratio with 95% Confidence interval**	**P value**
**CARRA-VID**	HR 1.53<br>95% CI (1.44-1.64)	<0.001 *
**CURB-65**	HR 1.83<br>95% CI (1.69-1.97)	<0.001 *
**CARRA-VID**	HR 1.45<br>95% CI (1.36-1.56)	<0.001 *
**4C mortality score**	HR 2.23<br>95% CI (1.97-2.52)	<0.001 *
**CARRA-VID**	HR 1.71<br>95% CI (1.59-1.83)	<0.001 *
**VACO index**	HR 1.18<br>95% CI (1.09-1.28)	<0.001 *
**CARRA-VID**	HR 1.75<br>95% CI (1.65-1.86)	<0.001 *
**WHO disease severity**	HR 2.32<br>95% CI (2.06-2.63)	<0.001 *

*Statistically significant at level P<0.05 / Four bivariable Cox regression models are presented, evaluating the prognostic properties of the CARRA-VID while simultaneously controlling for one other prognostic score. / Abbreviations: HR – hazard ratio, CI – confidence interval, CARRA-VID – CRP to Albumin Ratio, Red blood cell distribution width and Age based score for prognostication of hospitalized COVID-19 patients, CURB-65 – Confusion, Urea, Respiratory rate, Blood pressure and 65 years of age, VACO index – Veterans Health Administration COVID-19, WHO – World Health Organization.

Supplementary Table S4: Overview of the distribution of investigated variables used for the creation of the CARRA-VID score in the development cohort.

	Arithmetic mean ± standard deviation	Median and interquartile range
**CRP** (mg/L)	109.05 ± 86.66	91.1 (42.9-153.8)
**Albumin** (g/L)	31.43 ± 5.09	32 (28-35)
**CAR**	3.7 ± 3.17	2.91 (1.32-5.26)
**RDW** (%)	14.66 ± 2.08	14.1 (13.4-15.3)
**Age** (years)	71.1 ± 13.51	73 (64-81)

Abbreviations: CRP – C reactive protein, CAR – CRP to albumin ratio, RDW – red blood cell distribution width.

Supplementary Figure S5: Histograms of distribution of **A)** C reactive protein (CRP), **B)** albumin, **C)** CRP to albumin ratio (CAR), **D)** red blood cell distribution width (RDW) and **E)** age.

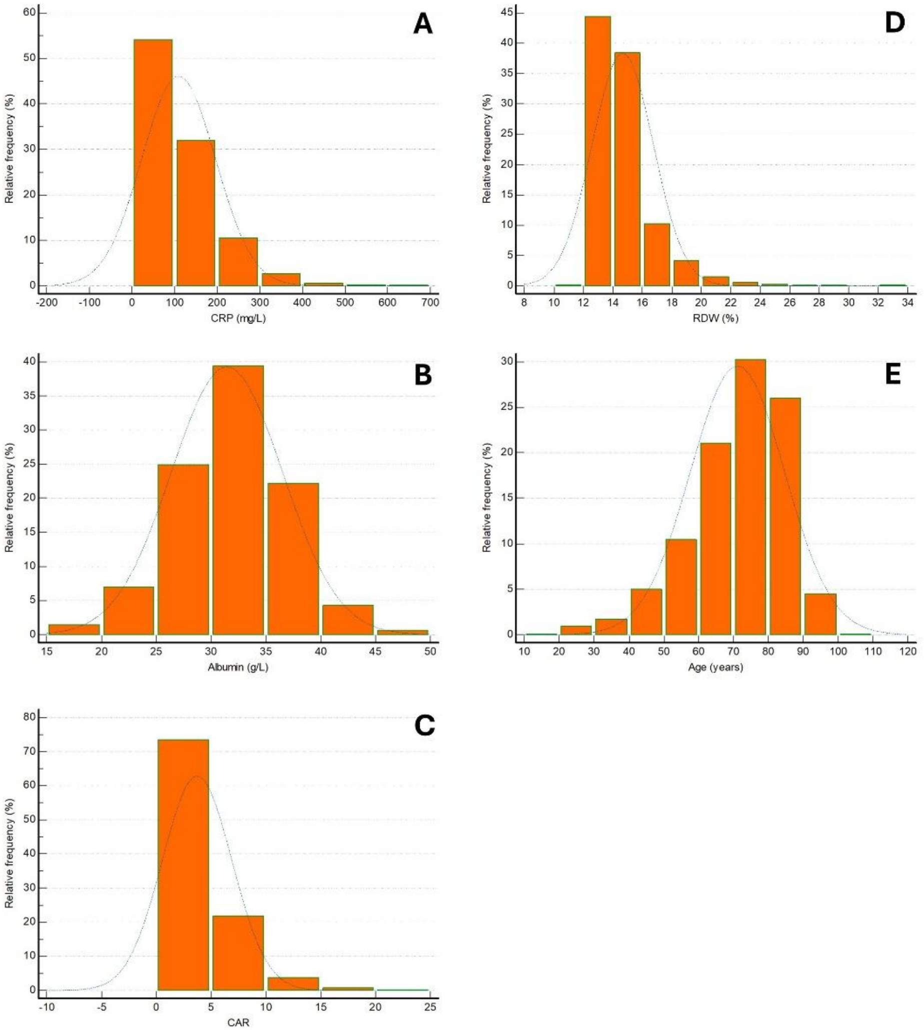

Supplement: Supplementary file 1 [file viruses-17-00629-s001.zip › viruses-3587946-Supplementary-S2.pdf]
